# Evaluation of the Nutritional Quality of the Five Commercial Fish Species From the Persian Gulf From Human Health Perspective

**DOI:** 10.1002/fsn3.70704

**Published:** 2025-07-26

**Authors:** Behrooz Mohammadzadeh, Javad Feizy

**Affiliations:** ^1^ Department of Fisheries, Faculty of Agriculture and Natural Resources Gonbad Kavous University Gonbad Kavous Iran; ^2^ Department of Food Safety and Quality Control Research Institute of Food Science and Technology (RIFST) Mashhad Iran

**Keywords:** fish healthy diet, nutritional value, omega‐3 fatty acids, Persian Gulf

## Abstract

This study aimed to assess the nutritional value of the five commercial fish species from the Persian Gulf. All species were low‐fat (0.07–1.64 g/100 g) and low‐calorie fish (108.54–126.17 cal/100 g). Lysine was the most important essential amino acid (EAA) (27.52%–28.82%). The total aromatic amino acids (TArAA), the ratios of essential amino acids to total amino acids (EAA/TAA), and the essential amino acids to non‐essential amino acids (EAA/NEAA) were higher than recommended by FAO/WHO. Tryptophan was the primary limiting amino acid. *Nemipterus japonicus* had the best protein quality with an EAAI (75.92) and BV (71.02). Oleic acid (57.32%–72.75%) and alpha‐linolenic acid (14.81%–39.45%) were the most abundant monounsaturated (MUFA) and polyunsaturated fatty acids (PUFA), respectively. The *Platycephalus indicus* , with high PUFA and PI levels (3.97% and 7.02%, respectively), has better lipid quality than other species. Consuming 111 g (59% meal portion), 258 g (138%), 180 g (96%), and 443 g (237%) of *Atropus atropos* , *Sphyraena jello* , *N. japonicus* , and *Argyrops spinifer* , respectively, could meet daily Ca, K, Fe, and Zn requirements. The Na/K ratio in five species and the Ca/Mg ratio in *A. atropos* and *N. japonicus* were in the standard range. In conclusion, all five fish species are good sources of lysine, oleic acid, alpha‐linolenic acid, K, Ca, Zn, and Fe. *N. japonicus* and *P. indicus* have the best protein and lipid nutritional quality, respectively; they can be suggested for a healthy diet.

## Graphical abstract

Based on the findings of this study, the best protein quality related to *N. japonicus* with an EAA index of 75.92. *P. indicus* has the best lipid quality due to the high ratio of ω‐3/ω‐6 (1.68) and PI (7.02). The abundant trace elements in all five fish species, K, Ca, Fe, and Zn, as well as these fish species, have good nutritional quality based on Na/K and Ca/K ratios.

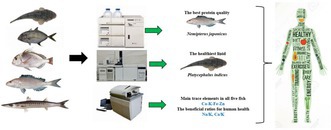

## 1 Introduction

Fish are nutritionally valuable due to their high‐quality proteins, beneficial lipids, and essential micronutrients (Khalili Tilami and Sampels 2018). The health effects of fish in humans are primarily due to the nutritional composition, including omega‐3 polyunsaturated fatty acids, balanced amino acids, and sufficient content of essential minerals such as calcium, phosphorus, iodine, selenium, iron, and zinc (Lund 2013). The price diversity of commercial fish has made this animal protein source accessible to various consumer groups with differing purchasing power (Mohanty et al. 2019). As seafood consumption continues to grow, FAO reports indicate the average annual per capita intake of fishery products reached 20.7 kg in 2022 (FAO 2024). With the increasing consumption of fish, understanding the nutritional composition of different fish species has become essential. The main reasons for this matter include maximizing the utilization of each species, the high biodiversity of fish, and differences in their nutritional compositions and nutritional quality (Mohanty et al. 2019). Numerous food composition databases have been established worldwide, such as the INFOODS database, managed by FAO, which documents the nutritional compositions of 526 fish species (FAO et al. 2017). Collecting and compiling data on the nutritional composition of various fish species has numerous applications, including promoting consumer awareness, enhancing the economic value of each fish species, calculating fish consumption levels for dietary planning, and providing essential data for nutritional research and public health studies (Greenfield and Southgate 2003; Mohanty et al. 2015; Rifat et al. 2023). The Persian Gulf, situated between 25°–30° N and 48°–56° E, is a semi‐enclosed marginal sea (Owfi et al. 2017). The volume of fisheries caught in the southern waters of Iran (The Persian Gulf and the Oman Sea) was 741,307 tons, equivalent to 52.3% of the total fisheries production of Iran (Iranian Fisheries Statistical Yearbook 2024). In the current study, some trace elements (Mn, Cu, Zn, Fe, Se, and Mo) can be considered heavy metals with contamination potential. Notably, these heavy metals have a significant environmental and public health concern in the coastal areas of Bushehr of the Persian Gulf (Mokarram et al. 2021; Malik and Muzaffar 2024). In the numerous published studies, the main pollution sources of heavy metals are petrochemical plants and industrial discharges, shipping and port activities in the Bushehr region of the Persian Gulf (Esmaeilbeigi et al. 2023; Torabi et al. 2024).

Many studies have already been conducted to determine and assess the nutritional composition of commercial fish in the Persian Gulf. Measurement of trace elements in six commercially important species, including fish ( *Euryglossa orientalis* , *Sardinella longiceps* , and *Carcharhinus dussumieri* ) (Soltani et al. 2021), evaluates nutritional values, including determination of proximate, fatty acid, and mineral composition in raw and cooked Orange‐spotted grouper ( *Epinephelus coioides* ) (Momenzadeh et al. 2017). The average concentration of trace metals (Cu, Zn, Cd, Pb, and Cr) in *Sphyraena jello* , *Nemipterus japonicus* , and *Argyrops spinifer* was suitable for human consumption and below the concentration proposed by WHO/FAO/USEPA (Mortazavi et al. 2024). *Atropus atropos* , as a commercial Carangidae, demonstrates notable omega‐3 fatty acid content of 0.314 g per 100 g of tissue (Hicks et al. 2019). Japanese threadfin bream ( *Nemipterus japonicus* ) has suitable omega‐3 fatty acid content (796.5 mg/100 g wet sample) and n‐3/n‐6 (5) (Abd Aziz et al. 2013).

Despite numerous studies about nutritional composition, there is limited comprehensive research assessing the nutritional quality of Persian Gulf commercial fish, particularly their amino acid, fatty acid, and mineral profiles, for designing healthy diets. Also, awareness of seafood's nutritional quality has grown among Iranians, as it is widely regarded as a healthful component of dietary planning. Additionally, assessing nutritional composition supports policy planning for dietary institutions and provides region‐specific data on Persian Gulf fish. Therefore, this study aims to evaluate the nutritional quality of five fish species from the North Persian Gulf that are commercially important and consumed by residents. These species include Cleftbelly trevally ( *Atropus atropos* ), Japanese threadfin bream ( *Nemipterus japonicus* ), Bartail flathead ( *Platycephalus indicus* ), King soldier bream ( *Argyrops spinifer* ), and Pickhandle Barracuda ( *Sphyraena jello* ).

## 2 Materials and Methods

### 2.1 Collection and Preparation of Fish Samples

**FIGURE 1:**
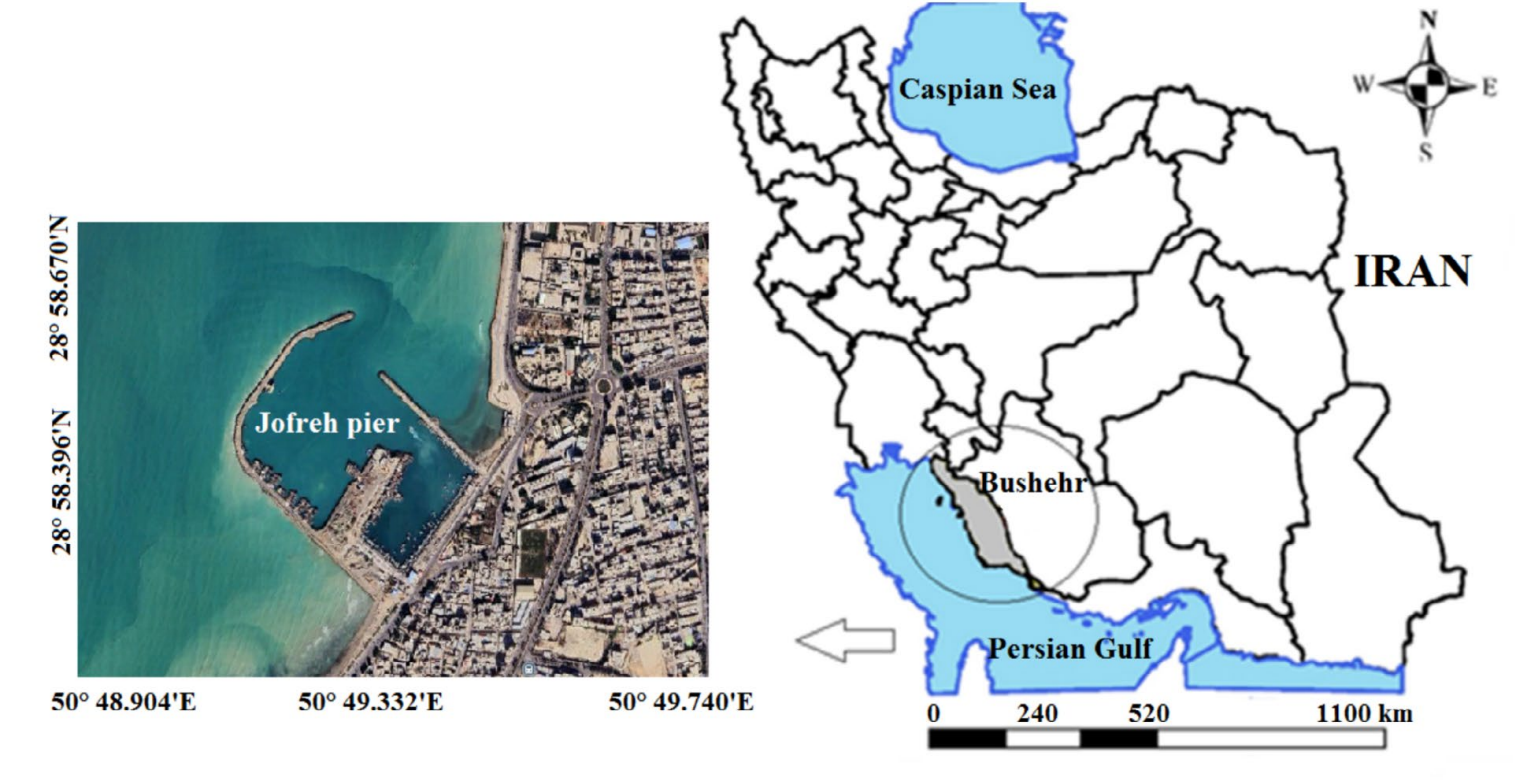
Geographic location of sampling site in Jofreh Pier, Bushehr province.

In this study, five commercial fish species were collected from Jofreh Pier as a fisheries pier in Bushehr province, where it is located in the northern part of the Persian Gulf during the catch season (autumn) (Figure 1). Table 1 presents an overview of the information on each species sampled, including the scientific name, English name, natural habitat, standard length (cm) and total weight (g). From each fish species, 10 specimens were collected. The samples, immediately after the catch, were stored in a refrigerated container, transported to the laboratory, and maintained at −20°C until examination. Fish biometry, including standard length and total weight, was measured in the lab following thawing at 25°C ± 2°C to prepare the sample for analysis. At first, the fish was gutted; then skin and bones were separated from the muscles manually, and then the muscle was homogenized using a meat grinder (Panasonic, MK‐ZJ2700, Japan). The homogenized meat muscle was used for the following assays. For all of the analyses, the number of analysis replications was 3.

**TABLE 1:** Information of fish samples (standard length (cm) and total weight (g)) in this study (mean ± SD; *n* = 3).

Scientific name	English name	Habitat	Standard length	Total weight
*Platycephalus indicus*	Bartail flathead	Benthic	29.95 ± 1.67	299.50 ± 53.62
*Atropus atropos*	Cleftbelly trevally	Pelagic	19.63 ± 1.30	215.86 ± 49.63
*Nemipterus japonicus*	Japanese threadfin bream	Benthic	21.55 ± 1.64	249.58 ± 57.91
*Argyrops spinifer*	King soldier bream	Benthic	18.08 ± 1.08	227.31 ± 32.12
*Sphyraena jello*	Pickhandle Barracuda	Pelagic	38.15 ± 1.18	438.00 ± 32.68

### 2.2 Determination of Proximate Composition and Total Energy

Ash and moisture contents were determined based on AOAC (2000b): method numbers (923.03 and 925.45, respectively) by weighing the samples before and after burning for 24 h at 550°C in an electric furnace for ash content and at 105°C in an oven for 24 h for moisture content. Total protein and crude lipid were measured using the Kjeldahl and Soxhlet apparatus and according to AOAC (2000a) method numbers (981.10 and 996.06, respectively). Carbohydrate and total energy were calculated using the following equations (Moniruzzaman et al. 2021):

$$\text{Carbohydrate}\;\left(\%\right)=100-\left(\text{Protein}\%+\mathrm{Fat}\%+\text{Moisture}\%+\mathrm{Ash}\%\right)$$

(1) $$\begin{array}{cc} \text{Total Energy}\;\left(\mathrm{KJ}/100\;g\right)= & 17.2\times \text{Carbohydrate}\;\left(g\right)+39.5\\ & \times \text{Lipid}\;\left(g\right)+23.6\times \text{Protein}\;\left(g\right) \end{array}$$

### 2.3 Amino Acid Analysis and Calculation of Protein Quality Indexes

For amino acid analysis, the samples were first prepared by hydrolyzing 0.2 g of fish muscle with 6 mol/L hydrochloric acid for 20 h at 110°C in a nitrogen environment. After hydrolysis, the sample was diluted using a borate solution (125 mM, pH 9.4). Then, 200 μL of borate solution, 800 μL of methanol (98%), and 50 μL of L‐homoserine solution (1 mmol/L) were added to a test tube and vortexed for 20 s. Next, 250 μL of the solution from the previous step, 100 μL of borate solution (125 mmol/L, pH 9.4), and 50 μL of OPA solution were added to another tube and vortexed for 2 min. Subsequently, 25 μL of 0.75 mol hydrochloric acid was added to the mixture. Finally, 50 μL of the resulting solution and 200 μL of borate solution (125 mmol/L, pH 9.4) were mixed for several seconds and then injected into a high‐performance liquid chromatography (HPLC) system equipped with a fluorescent LC350 detector (Young Lin, South Korea). The HPLC system consists of a C18 column with dimensions of 15 cm length, 4.6 mm diameter, and 5 μm particle size, with a temperature of 35°C. The flow rate of the mobile phase was 1.3 mL/min. The concentration of total amino acids was expressed in g per 100 g (dry weight sample). The amino acid score, chemical score, essential amino acid index, biological value, and protein performance indicators were calculated based on the following equations (Oztekin et al. 2020). To assess the nutritional value availability, firstly, the amino acid content of the fish protein was calculated by multiplying the amino acid amount by 0.625 (Oztekin et al. 2020):

(2) $$\text{Amino acid concentration}\;\left(\mathrm{per}\;\text{fish protein}\right)=\left(\text{essential amino acid}\right)\times 0.625$$

(3) $$\text{Amino acid score}=\left(\text{amino acid concentration}/\text{amino acid standard}\right)$$

(4) Chemical score=amino acid concentration/amino acid concentration ineggprotein

(5) $$\text{Essential amino acid index}=\;\sqrt[{n}]{\left[\left(\frac{{\mathrm{EAA}}_{1}\times 100}{{\mathrm{EAA}}_{1\mathrm{EG}}}\;\right)\times \left(\frac{{\mathrm{EAA}}_{2}\times 100}{{\mathrm{EAA}}_{2\mathrm{EG}}}\right)\times \left(\frac{{\mathrm{EAA}}_{n}}{{\mathrm{EAA}}_{n\mathrm{EG}}}\right)\right]}$$

(6) $$\text{Biological value}=\left(\text{essential amino acid index}\times 1.09\right)-11.73$$

(7) $$\text{Protein efficiency}=-0.468+0.454\times \left(\text{leucine}\right)-0.105\times \left(\text{tyrosine}\right)$$

### 2.4 Fatty Acid Analysis and Lipid Quality Indexes

Methanol and chloroform were used for the solvent extraction of fish oil from muscle (Folch et al. 1957). Then, the extracted oil is converted to methyl ester form using boron trifluoride, hexane, and saturated brine. Fatty acids were analyzed using a gas chromatographic (PHILIPS, Netherlands) system equipped with a BPX70 column (60 m × 0.32 mm ID × 0.25 μm), split injector, and a flame ionization detector. The carrier gas was helium, with a flow rate of 1 mL/min. The temperature of the inlet and detector was 250°C. Fatty acid content was expressed in the percentage of total fatty acids. The quality of fish muscle lipid was evaluated using quality indices after determining its fatty acid content. The Ulbricht and Southgate (1991) formula was used to calculate the thrombogenic index (TI) and the atherogenic index (AI). The hypocholesterolemic to hypercholesterolemic ratio (HH) was calculated according to the Santos‐Silva et al. (2002) method. The fatty acid prooxidant index (PI) was measured based on Arakawa and Sagai (1986), and the Karsli (2021) formula was used to determine the health‐promoting index (HPI). AI, TI, HH, PI, and HPI were calculated based on the following equations.

(8) $$\mathrm{AI}=\left[\left(C12:0+\left(4\times C14:0\right)+C16:0\right)\right]/\left[\Sigma \text{MUFA}+\Sigma \left(n‐6\right)+\Sigma \left(n‐3\right)\right]$$

(9) $$\left(13\right)\mathrm{TI}=\left(C14:0+C16:0+C18:0\right)/\left[0.5\times \Sigma \text{MUFA}+0.5\times \Sigma \left(n‐6\right)+3\times \Sigma \left(n‐3\right)+\mathrm{\Sigma n}‐3/\mathrm{\Sigma n}‐6\right]$$

(10) $$\mathrm{HH}=\left(C18:1n‐9+C18:2n‐6+C20:4n‐6+C18:3n‐3+C22:5n‐3+C22:6n‐3\right)/\left(C14:0+C16:0\right)$$

(11) $$\mathrm{PI}=\left(\%\text{monoenoic}\times 0.025\right)+\left(\%\text{dienoic}\times 1\right)+\left(\%\text{trienoic}\times 2\right)+\left(\%\text{tetraenoic}\times 4\right)+\left(\%\text{pentaenoic}\times 6\right)+\left(\%\text{hexaenoic}\times 8\right)$$

(12) $$\mathrm{HPI}=\text{ƩMUFA}/\left[C12:0+\left(4\times C14:0\right)+C16:0\right]$$

### 2.5 Trace Element Determination

The homogenized muscle was dried in an oven for 48 h at a temperature of 70°C. The dried materials were wet‐digested using 65% nitric acid under a fume hood. Subsequently, the solution was filtered using filter paper number 42 and then diluted to a volume of 25 mL using distilled water. Finally, the trace elements concentration was determined using an Inductively coupled plasma mass spectrometry device (ICP‐MS ELAN 9000, USA), and macro elements and microelements were reported in mg/kg and μg/g (ppm) dry weight of samples, respectively (Kazemi et al. 2022). The main ratios between *Ca/P*, *Ca/Mg*, *Ca/K*, and *Na/K* were also calculated (Adeyeye et al. 2019; Shi et al. 2020).

### 2.6 Quality Control and Quality Assurance

High‐purity laboratory‐grade compounds and reagents were used throughout the study. To ensure the absence of contaminants, all analytical vessels were thoroughly cleaned with ultrapure HNO_3_, followed by rinsing with deionized water. All chemical reagents were of analytical grade and underwent additional purification as needed. To minimize contamination risks during sample preparation and analysis, all glassware was meticulously washed and rinsed with ultrapure acid and deionized water. Additionally, deionized ultrapure water was used to prepare all calibration standards and additional reagents. A standard stock solution of 10–100 ppm for all the metals was used, which was obtained from Merck (Darmstadt, Germany) (ICP multi‐element standard solution VI). Amino acid standards were obtained from Sigma‐Aldrich (St. Louis, MO, USA). Fatty acid methyl ester standards were purchased from Supelco (Bellefonte, PA, USA). Analyses were performed in triplicate to ensure reproducibility. Additionally, spike recovery tests were performed for trace elements, amino acids, and fatty acids to validate methodological precision and accuracy.

### 2.7 Statistical Data Analysis

The statistical analysis was carried out using SPSS 21 software. The Shapiro–Wilk test was used to determine the normality of data. Also, one‐way analysis of variance (ANOVA) was employed to determine differences in fatty acid, amino acid, and lipid quality indices content among the five fish species' muscles. Duncan's multiple range test was used to compare the means. A significance threshold of *p*‐value = 0.05 was applied to each statistical test.

## 3 Results and Discussion

### 3.1 Proximate Composition Total Energy

The proximate composition of fish muscle is shown in Table 2. No significant differences existed between the protein content of different studied species (*p* < 0.05). *A. spinifer* and *N. japonicus* had the highest (1.64 g/100 g) and lowest (0.07 g/100 g) lipid contents, respectively. *N. japonicus* and *S. jello* had the highest (81.49 g/100 g) and lowest (76.52 g/100 g) moisture, respectively. *A. spinifer* and *P. indicus* had the highest (1.88 g/100 g) and lowest (1.06 g/100 g) ash content, respectively. *A. spinifer* and *N. japonicus* had the highest (528 kJ/100 g) and lowest (454 kJ/100 g) energy levels. The protein and lipid content of fish muscle is influenced by the fish's diet, body weight, environment, fishing season, intensity of feeding, and quantity of natural food consumed (Pyz‐Łukasik et al. 2020). The minimum muscle lipid levels in *Nemipterus japonicus* can be attributed to some parameters, such as the development and maturation of the gonads in fish, which lead to a decline in the crude lipids of muscle (Sheridan et al. 1983). Also, high tolerance was observed in lipid content for *Nemipterus japonicus* (14.8% to 19.3% in the study of El‐Halfawy and Amadan 2015 and 21.39% in the study of Gull et al. 2024). This study's range of lipid (0.07–1.64 g/100 g) and protein (17.49–20.57 g/100 g) is consistent with other studies. For example, Usydus et al. (2011) and Karl et al. (2014) reported that the low and high protein content (18.9% and 12.2%) for trout and walleye pollock, respectively. Also, Karl et al. (2014) reported that low and high lipids (0.8% and 3.4%) for *Rachycentron canadum* and *Macruronus magellanicus* , respectively. According to Pyz‐Łukasik et al. (2020), fish are classified based on lipid content as lean (up to 2%), medium‐fat (2%–7%), fatty (7%–15%), or high‐fat (over 15%). Therefore, all five fish species studied in the present work are classified as lean fish. Analysis of *Epinephelus fuscoguttatus* as a whole fish showed a high ash content (25.44%) (Ali et al. 2020). The particular kind of examined tissue can have a huge impact on the results of chemical composition analysis. As fish bones contain a substantial amount of calcium and phosphorus, the lower ash content (1.06–1.88 g/100 g) likely resulted from analyzing skinless, boneless muscle (Malde et al. 2010). It is well known that fish is low in calories; even fatty fish have fewer calories than red meat (Alkuraieef et al. 2021). The range of total energy values in this study was 452 to 472 kJ/100 g, equivalent to 108.54 to 126.17 cal, which is less than the range of 199–200 cal per 100 g reported for most finfish and seafood (Mohamed and El Lahamy 2020). Therefore, these fish can be suggested as a low‐calorie food alternative in dietary regimens.

**TABLE 2:** Proximate composition (includes protein, lipid, ash and moisture as g/100 g) and total energy (Kj/100 g) of Bartail flathead ( *Platycephalus indicus* ), Cleftbelly trevally ( *Atropus atropos* ), Japanese threadfin bream ( *Nemipterus japonicus* ), King soldier bream ( *Argyrops spinifer* ) and Pickhandle Barracuda ( *Sphyraena jello* ), (mean ± SD; *n* = 3).

Proximate composition	*Platycephalus indicus*	*Atropus atropos*	*Nemipterus japonicus*	*Argyrops spinifer*	*Sphyraena jello*
Protein	20.57 ± 0.21^a^	17.49 ± 2.22^a^	19.13 ± 0.55^a^	19.43 ± 3.16^a^	20.30 ± 0.66^a^
Lipid	0.44 ± 0.02^b^	0.30 ± 0.02^b^	0.07 ± 0.01^c^	1.64 ± 0.19^a^	0.31 ± 0.03^b^
Moisture	79.83 ± 0.5^b^	78.39 ± 0.35^c^	81.49 ± 0.33^a^	78.68 ± 0.25^c^	76.52 ± 0.98^d^
Ash	1.06 ± 0.07^d^	1.22 ± 0.1^cd^	1.59 ± 0.17^b^	1.88 ± 0.10^a^	1.32 ± 0.05^c^
Energy	502.62 ± 5.60^ab^	472.14 ± 11.55^ab^	454.44 ± 13.45^b^	528.26 ± 76.56^a^	518.04 ± 17.49^ab^

*Note:* Different superscript letters (a–d) in the same row for proximate composition represent significant differences (*p* ≤ 0.05) among different fish species.

### 3.2 Amino Acid Profile, Groups, and Ratios

Table 3 presents the amino acid profile, groups, and ratios of the studied fish muscles. Lysine was the predominant essential amino acid in all species. The *N. japonicus* has the most excellent lysine content (12.37 g/100 sample). Glutamic acid was the most common non‐essential amino acid in all five fish species, and among them, *N. japonicus* had the most excellent glutamic acid concentration (17.50 g/100 g). Glutamic acid is an essential nitrogen source. It plays a pivotal role in amino acid metabolism because it is essential for enzyme solubility and keeping ionic properties during enzyme action (Özden and Erkan 2008). The frequency of glutamic acid and lysine in marine fish has been reported in multiple studies. Similarly, Taheri et al. (2018) reported that the glutamic acid and lysine content of *Sphyraena jello* from the Oman Sea were 11.17 and 8.40 g/100 g, respectively. The total amino acid content (TAA) of *N. japonicus* muscle was significantly higher (*p* ≤ 0.05) than other fish species (Table 2). Cobas et al. (2022) reported that the TAA content in swordfish ( *Xiphias gladius* ) muscle was 17.49 g/100 g. The amino acid content of fish species from the Baltic Sea, including cod, herring, and salmon, varied from 43.7 to 44.2 g/100 g of protein (Usydus et al. 2011). In this study, the TAA varied from 69.85 to 94.87 g/100 g, which is substantially higher than the TAA of marine fish muscles that have previously been reported. Good protein quality is confirmed by the presence of essential amino acids at enough levels in the diet (Mahmud et al. 2020). *N. japonicus* muscle had significantly higher total essential amino acid content (42.91 g/100 g) compared to other fish species (*p* ≤ 0.05). Among the various amino acid categories, non‐essential amino acid (NEAA)was the most abundant of all five species. The muscle of *N. japonicus* exhibited a substantially higher amount of (51.95 g/100 g) compared to other fish species (*p* ≤ 0.05). NEAA, like glycine, proline, glutamine, and aspartic acid, have cytotoxic effects on cancer cells and also could improve and accelerate wound healing (Kim et al. 1999). Compared to other species, *N. japonicus* exhibited a substantially higher total basic amino acid (BAA) content (19.56 g/100 g) and acidic amino acid (AAA) content (58.28 g/100 g) (*p* ≤ 0.05). In the present study, except for *A. atropos* , BcAA contents of all species ranged from 12.65 to 17.47 g/100 g, higher than the required standard value. FAO/WHO/UNU (2007) has set a standard value of 12.8 g/100 g of protein for total branched‐chain amino acids (BcAA) that consist of leucine, isoleucine, and valine. In the present study, the total aromatic amino acids (ArAA), which include phenylalanine and tyrosine, content of the studied fish ranged from 5.80 to 7.67 g/100 g, which is higher than the FAO/WHO standard value (0.3 g/100 g, FAO/WHO/UNU 2007). Fish flavor and taste qualities are linked to amino acid components such as alanine, aspartic acid, glutamic acid, and glycine (Ruiz‐Capillas and Moral 2004). *N. japonicus* muscle had a significantly higher total level of flavor‐enhancing amino acids (39.31 g/100 g) than other fish species (*p* ≤ 0.05). The five species of the current study had SAA concentrations ranging from 1.79 to 2.28 g/100 g. Between the studied fish in the present work, the SSA content of *A. spinifer* and *N. japonicus* was higher than the standard level (2.2 g/100 g) (FAO/WHO/UNU 2007). FAO/WHO (1991) recommends a ratio of 40% for EAA/TAA. In our study, this ratio ranged from 44.47% to 47.46%, which implies the high protein quality of the studied fish. The ratio EAA/NEAA is employed as a measure of protein quality (Cobas et al. 2022). FAO/WHO (1991) advised a ratio of 60% for EAA/NEAA. The high protein quality of the muscle in all fish was confirmed by the high ratio of EAA/NEAA (80.19% to 90.36%), which is higher than the FAO/WHO recommended value. The Leu/Ile ratio was low, ranging from 1.76% to 1.96%, and there was no statistically significant difference between the five fish species (*p* ≥ 0.05). Similarly, Taheri et al. (2018) reported that this ratio in the *Sphyraena jello* from the Oman Sea was low (1.14%). Spawning season, species, size, food, and age can affect an organism's amino acid content (Duyar and Eke 2009). The leucine/isoleucine (Leu/Ile) ratio balance is more important than leucine alone, especially when controlling the development metabolism of tryptophan and niacin and receiving attention (Adeyeye 2009).

**TABLE 3:** Amino acid profile (g/100 g DW), groups, and ratios in the fish muscle of Bartail flathead ( *Platycephalus indicus* ), Cleftbelly trevally ( *Atropus atropos* ), Japanese threadfin bream ( *Nemipterus japonicus* ), King soldier bream ( *Argyrops spinifer* ), and Pickhandle Barracuda ( *Sphyraena jello* ), (mean ± SD; *n* = 3).

Amino acid	*Platycephalus indicus*	*Atropus atropos*	*Nemipterus japonicus*	*Argyrops spinifer*	*Sphyraena jello*
Histidine	2.18 ± 0.15^a^	1.62 ± 0.28^b^	1.94 ± 0.05^ab^	2.05 ± 0.29^a^	1.89 ± 0.19^ab^
Isoleucine	4.12 ± 0.19^ab^	3.15 ± 0.14^d^	4.45 ± 0.46^a^	3.91 ± 0.11^bc^	3.53 ± 0.21^cd^
Leucine	7.22 ± 0.37^b^	6.15 ± 0.41^b^	8.50 ± 1.16^a^	7.44 ± 0.35^ab^	6.77 ± 0.64^a^
Lysine	11.17 ± 0.32^b^	8.55 ± 0.30^d^	12.37 ± 0.81^a^	10.85 ± 0.21^b^	9.81 ± 0.83^c^
Methionine	2.16 ± 0.27^ab^	1.96 ± 0.07^bc^	2.27 ± 0.05^a^	2.28 ± 0.11^a^	1.79 ± 0.08^c^
Phenylalanine	3.80 ± 0.22^a^	2.81 ± 0.02^b^	3.87 ± 0.13^a^	3.85 ± 0.23^a^	3.24 ± 0.42^b^
Tryptophan	0.69 ± 0.14^ab^	0.48 ± 0.07^c^	0.56 ± 0.08^bc^	0.80 ± 0.14^a^	0.44 ± 0.06^c^
Threonine	3.92 ± 0.10^b^	2.99 ± 0.12^d^	4.43 ± 0.43^a^	3.75 ± 0.09^bc^	3.32 ± 0.34^cd^
Valine	4.21 ± 0.29^a^	3.35 ± 0.07^b^	4.52 ± 0.30^a^	4.50 ± 0.09^a^	3.71 ± 0.34^b^
Alanine	5.37 ± 0.27^b^	4.75 ± 0.30^b^	6.21 ± 0.59^a^	5.28 ± 0.16^b^	4.64 ± 0.64^b^
Arginine	4.86 ± 0.25^a^	4.07 ± 0.28^b^	5.24 ± 0.38^a^	4.74 ± 0.18^a^	3.92 ± 0.42^b^
Aspartic acid	8.74 ± 0.52^bc^	7.71 ± 0.34^c^	11.08 ± 1.13^a^	9.54 ± 0.35^b^	8.35 ± 0.76^bc^
Glutamic acid	13.73 ± 0.54^bc^	12.42 ± 0.71^c^	17.50 ± 0.97^a^	14.52 ± 0.30^b^	12.69 ± 1.28^c^
Glycine	3.62 ± 0.20^bc^	4.03 ± 0.41^ab^	4.52 ± 0.24^a^	3.91 ± 0.35^b^	3.25 ± 0.23^c^
Serine	3.52 ± 0.35^ab^	2.82 ± 0.09^c^	3.84 ± 0.10^a^	3.58 ± 0.21^ab^	3.21 ± 0.27^c^
Tyrosine	3.87 ± 0.10^a^	2.99 ± 0.43^b^	3.57 ± 0.37^a^	2.87 ± 0.07^b^	2.81 ± 0.17^b^
Ʃ TAA	83.19 ± 0.79^b^	69.85 ± 1.11^c^	94.87 ± 5.78^a^	83.88 ± 1.46^b^	73.37 ± 3.85^c^
Ʃ EAA	39.48 ± 0.20^b^	31.05 ± 0.60^d^	42.91 ± 2.19^a^	39.43 ± 1.45^b^	34.50 ± 2.62^c^
Ʃ NEAA	43.71 ± 0.99^b^	38.79 ± 1.71^c^	51.95 ± 3.59^a^	44.45 ± 0.03^b^	38.87 ± 1.23^c^
Ʃ BcAA	15.55 ± 0.12^b^	12.65 ± 0.34^c^	17.47 ± 1.00^a^	15.85 ± 0.56^b^	14.01 ± 1.19^c^
Ʃ ArAA	7.67 ± 0.32^a^	5.80 ± 0.41^d^	7.44 ± 0.51^ab^	6.73 ± 0.30^bc^	6.05 ± 0.59^cd^
Ʃ DAA	31.47 ± 0.50^bc^	28.91 ± 1.09^c^	39.31 ± 2.94^a^	33.26 ± 0.46^b^	28.93 ± 0.92^c^
Ʃ BAA	18.21 ± 0.42^ab^	14.24 ± 0.30^c^	19.56 ± 1.13^a^	17.64 ± 0.68^b^	15.62 ± 1.06^c^
Ʃ AAA	22.48 ± 0.03^bc^	20.13 ± 0.37^d^	28.58 ± 2.11^a^	24.06 ± 0.65^b^	21.04 ± 0.51^cd^
Ʃ SAA	2.16 ± 0.27^ab^	1.96 ± 0.07^bc^	2.27 ± 0.05^a^	2.28 ± 0.11^a^	1.79 ± 0.08^c^
EAA/TAA	47.46^a^	44.47^c^	45.25^bc^	47.00^ab^	46.98^ab^
NEAA/TAA	52.54^c^	55.53^a^	54.75^ab^	53.00^bc^	53.02^bc^
EAA/NEAA	90.36^a^	80.19^c^	82.67^bc^	88.71^ab^	88.67^ab^
DAA/TAA	37.82^c^	41.38^a^	41.42^a^	39.66^b^	39.46^b^
Leu/Iso	1.76^a^	1.96^a^	1.94^a^	1.90^a^	1.92^a^

*Note:* Different superscript letters (a–d) in the same row represent significant differences (*p* ≤ 0.05) among different fish species.

### 3.3 Protein Quality Indexes Based on Amino Acid Analysis

Table 4 presents the EAAs concentration, amino acid content, chemical score, essential amino acid index (EAAI), biological value (BV), and protein efficiency ratio (PER) in the muscle protein of the studied fish. In comparison to the FAO/WHO standard, the muscle protein of *P. indicus* , *N. japonicus* , and *A. spinifer* has higher concentrations of lysine, threonine, and the sum of phenylalanine and tyrosine. Conversely, in the muscle protein of *A. atropos* and *S. jello* , only lysine and threonine were more than the FAO/WHO standard. Thus, the muscle protein quality of *P. indicus* , *N. japonicus* , and *A. spinifer* was higher than that of *A. atropos* and *S. jello* . The amounts of threonine were higher in the muscle of all fish compared to the protein egg. All fish muscle proteins had a lower EAA content than the FAO/WHO standard and the reference protein eggs. The amino acid score, chemical score, EAAI, BV, and PER ratio are the most crucial factors in determining the nutritional quality of proteins (Oztekin et al. 2020; Cobas et al. 2022). Low chemical scores indicate that insufficient amino acid content is lower than the human body needs (Oztekin et al. 2020). In addition, the priority of limiting amino acids in the protein composition is identified by analysis of the amino acid score and the chemical score (Pyz‐Łukasik and Paszkiewicz 2018; Gómez‐Limia et al. 2021). In this study, tryptophan was the first limiting essential amino acid in the muscle protein of all five fish species (Table 4). Indeed, based on the obtained results, the muscles of the studied species could not provide a human need for tryptophan. Similarly, the obtained result in the current study shows that the amino acid profile of *Sphyraena jello* included the least amount (0.04 g/100 g) of tryptophan (Taheri et al. 2018). The amino acid threonine had the most outstanding chemical and amino acid scores, indicating all five species' muscles could supply human demand for this vital amino acid. Threonine helps to preserve bone and teeth strength, enhance wound healing, and lessen fat accumulation in the liver. In addition, it supports the growth of the thymus, the heart and blood vessels, the liver, the central nervous system, and the immune system (Sarojnalini and Hei 2019). Threonine amino acid score has also been found to be high in a few marine fish, including *Pagellus acarne* (5.95) (Oztekin et al. 2020) and *Xiphias gladius* (1.82) (Cobas et al. 2022). Among the essential amino acids in *Pagellus acarne* , threonine got the highest chemical score (Oztekin et al. 2020). Like the chemical score, EAAI is calculated by comparing the essential amino acid composition of the protein with the standard protein and considers the presence of all essential amino acids needed for protein synthesis (Pyz‐Łukasik and Paszkiewicz 2018). If the EAAI is more than 90, the protein quality is ranked as high, while if the EAAI is less than 70, the protein quality is considered insufficient (Mir et al. 2019). In the current study, the EAAI was less than 90 in all species, and it was less than 70 for *S. jello* and *A. atropos* , indicating the protein quality of these two species is low. Consistent with our findings, the EAAI in *Pagellus acarne* was less than 90 and was 70.27 (Oztekin et al. 2020). However, higher EAAI values were reported for *Xiphias gladius* (146.11) and *Anguilla anguilla* (101.44) which shows their high protein quality (Gómez‐Limia et al. 2021; Cobas et al. 2022). Similarly, the PER is one of the most crucial metrics for determining the total nutritional quality of proteins (Cobas et al. 2022). If the predicted protein efficiency ratio of foods is less than 1.5, between 1.5 and 2, and more than 2, these foods are considered as foods with low, moderate, and high protein quality, respectively (Zengin et al. 2012). In the current study, PER values of all studied fish were higher than 2, so they are categorized as high‐protein quality foods. Another metric used to assess the nutritional quality of protein is BV, which is influenced by the amino acid composition (Shi et al. 2020). *N. japonicus* had the highest BV (71.02%) while *A. atropos* had the lowest (51.23%). The BV of *Pagellus acarne* was 64.86% (Oztekin et al. 2020). It is well known that biological value (BV) reflects the proportion of absorbed protein incorporated into human body proteins and correlates with EAAI (Shi et al. 2020). This relationship was confirmed in this study by high and low amounts of EAAI in *N. japonicus* and *A. atropos* (75.92 and 57.77, respectively). Also, based on BV, the protein of *N. japonicus is* a good source for supplying essential amino acids in the diet of humans.

**TABLE 4:** Above, Comparison of essential amino acid concentration in the five fish species' muscle and reference standards. Under: Comparison analysis of amino acid, chemical score, essential amino acid index (EAAI), biological value (BV), and protein efficiency (PE) in the fish muscle of Bartail flathead ( *Platycephalus indicus* ), Cleftbelly trevally ( *Atropus atropos* ), Japanese threadfin bream ( *Nemipterus japonicus* ), King soldier bream ( *Argyrops spinifer* ), and Pickhandle Barracuda ( *Sphyraena jello* ).

	Amino acid/species	*Platycephalus indicus*	*Atropus atropos*	*Nemipterus japonicus*	*Argyrops spinifer*	*Sphyraena jello*	FAO/WHO standard	Egg standard
Amino acid concentration	Histidine	1.36^a^	1.01	1.22	1.28	1.18	1.5	2.2
	Isoleucine	2.57	1.97	2.78	2.44	2.21	3	5.4
	Leucine	4.51	3.85	5.31	4.65	4.23	5.9	8.6
	Lysine	6.98	5.34	7.73	6.78	6.13	4.5	7
	Methionine	1.35	1.22	1.42	1.43	1.12	1.6	^b^
	Methionine + cysteine	1.35	1.22	1.42	1.43	1.12	2.2	5.7
	Phenylalanine + tyrosine	4.79	3.62	4.65	4.20	3.78	3.8	9.3
	Tryptophan	0.43	0.30	0.35	0.50	0.28	2.3	4.7
	Threonine	2.45	1.87	2.77	2.35	2.08	0.6	1.7
	Valine	2.63	2.09	2.83	2.81	2.32	2.9	6.6
	ƩEAA	11.75	9.23	12.60	11.46	9.98	28.3	51.2

Amino acid/species	Amino acid score					Chemical score				
	*Platycephalus indicus*	*Atropus atropos*	*Nemipterus japonicus*	*Argyrops spinifer*	*Sphyraena jello*	*Platycephalus indicus*	*Atropus atropos*	*Nemipterus japonicus*	*Argyrops spinifer*	*Sphyraena jello*
Histidine	0.91	0.67	0.81	0.85	0.79	0.62	0.46	0.55	0.58	0.53
Isoleucine	0.86	0.66	0.92	0.81	0.74	0.48	0.36	0.51	0.45	0.41
Leucine	0.76	0.65	0.90	0.79	0.72	0.53	0.45	0.62	0.54	0.49
Lysine	1.55	1.19	1.72	1.51	1.36	1.00	0.76	1.10	0.97	0.87
Methionine	0.85	0.77	0.89	0.89	0.70	^b^	^b^	^b^	^b^	^b^
Methionine + cysteine	0.62	0.56	0.64	0.65	0.51	0.24	0.21	0.25	0.25	0.20
Phenylalanine + tyrosine	1.26	0.95	1.23	1.11	0.99	0.52	0.39	0.50	0.45	0.41
Tryptophan	0.19	0.13	0.15	0.22	0.12	0.09	0.06	0.07	0.11	0.06
Threonine	4.08	3.12	4.61	3.91	3.46	1.44	1.10	1.63	1.38	1.22
Valine	0.68	0.54	0.73	0.72	0.59	0.40	0.32	0.43	0.43	0.35
EAAI	74.06	57.77	75.92	73.95	61.56					
BV	69.00	51.23	71.02	68.87	55.37					
PE	2.40	2.01	3.02	2.62	2.77					

^a^ Conversion of amino acid concentration in fish muscle protein via has been multiplied essential amino acid in Table 3 to a 0.625 coefficient. Essential amino acid content of FAO/WHO standard (FAO/WHO/UNU 2007; FAO/WHO 2003) and egg protein standard as mg per 100 g protein.

^b^ Not reported.

### 3.4 Fatty Acid Profile and Lipid Quality

Table 5 displays the fatty acid composition (FA) and associated indices in the fish muscle. Palmitic acid (C16:0) was the most prevalent saturated fatty acid (SFA) in the fish muscle. *A. atropos* had a significantly higher concentration of palmitic acid (50.43%) than the other species (*p* ≤ 0.05). In the loin of yellowfin tuna ( *Thunnus albacares* ) from the Atlantic, Indian, and Pacific Oceans ( *Anguilla anguilla* ), the most prevalent SFA was palmitic acid (C16:0), 23.1%, 29.3%, and 28.6% respectively (Domingues et al. 2021). Similarly, palmitic acid content as the main SFA in some small pelagic fishes, including *Cololabis saira* , *Decapterus macarellus* , *Engraulis japonicus* , *Entrumeus teres*, *Sardinops melanostictus* , *Scomber australasicus* , *Scomber japonicus* , and *Trachurus japonicus* was 15.40%, 16.37%, 21.37%, 22.48%, 22.78%, 17.19%, 18.20%, and 21.75% respectively (Ohshimo et al. 2023). Palmitic acid (C16:0) and stearic acid (C14:0), as SFAs, elevate blood cholesterol by impairing LDL receptor function (Feingold 2024). As a result, their elevated fish lipid levels suggest a possible danger of elevated cholesterol for human consumers. In this regard, among the five species, the lowest and highest sum of palmitic and stearic acid was 27.4% and 57.57% in *Argyrops spinifer* and *A. atropos*, respectively. This result shows that the lipids of *A. atropos* had a higher risk of increasing cholesterol than other species for human consumers. The most prevalent MUFA was oleic acid, with the lowest and highest oleic acid found in *N. japonicus* (8.64%) and *S. jello* (25.34%), respectively. Oleic acid (C18:1n‐9 cis), by increasing LDL receptor activation, causes a decrease in blood cholesterol levels (Chen and Liu 2020). Oleic acid in many freshwater and marine fish species is considered the main MUFA (Özogul et al. 2007). In golden gray mullet ( *Liza aurata* ) and gold stripe goatfish ( *Mulloidichthys vanicolensis* ), oleic acid was the most dominant MUFA, which ranged from 11.42% to 15.03% and 13.02% to 15.03% of total fatty acids, respectively (Küçükgülmez et al. 2018). Alpha‐linolenic acid was the most predominant PUFA in the muscle of all fish species. *P. indicus* had a significantly higher level of PUFA (2.45%) in its muscle than other species (*p* ≤ 0.05). In addition, the lowest alpha‐linolenic acid was in *N. japonicus* (0.20%). Fish's diet affects the amount of linoleic acid; so, fish that feed on zooplankton, tiny fish, or insects usually have high levels of this acid (Salimon and Rahman 2008). Compared to this study, the most dominant PUFAs in the muscle of *Cololabis saira* , *Decapterus macarellus* , *Engraulis japonicus* , *Entrumeus teres*, *Sardinops melanostictus* , *Scomber australasicus* , *Scomber japonicus* , and *Trachurus japonicus* were DHA in the range of 25.35% to 45.44% (Ohshimo et al. 2023). The most substantial PUFAs in *Liza aurata* and *Mulloidichthys vanicolensis* were arachidonic (C20:4n6) acid, EPA, and DHA (Küçükgülmez et al. 2018). Levels of EPA, DHA, and arachidonic acid are controlled by a variety of factors, including species, environment, diet, season, and water temperature (Pyz‐Łukasik et al. 2020). Unfortunately, DHA and EPA were undetectable in all five species. Since the conversion of alpha‐linolenic acid to EPA and DHA is insufficient in the human body, the intake of these main fatty acids from food sources is essential (Ganesan et al. 2014). Based on the findings of this study, none of the five species can supply this demand for humans. The ω‐3 (2.45%), ω‐6 (1.52%), and PUFA (3.97%) in *P. indicus* and SFA (87.9%) in *N. japonicus* were significantly higher than those of the other species (*p* ≤ 0.05). *S. jello* muscle had a significantly higher MUFA (38.26%) compared to the other species (*p* ≤ 0.05). Based on these, the most prevalent fatty acid groups of the studied fish in the current study were omega‐3, omega‐6, SFA, and MUFA. Consistent with these findings, SFA, MUFA, omega‐3, and omega‐6 contents of common dentex ( *Dentex dentex* ) were 37.1%, 34.8%, 14.1%, and 2.98%; for annular sea bream ( *Diplodus annularis* ) was 38.4%, 31%, 12.7%, 3.9%; for sheep head bream ( *Diplodus puntazzo* ) was 34%, 35%, 20.8%, 6.5%; in sea bream ( *Diplodus sargus* ), 37.7%, 25.4%, 16.4%, 7.59%; and for sea bream ( *Diplodus vulgaris* ) 37.2%, 25.2%, 27.1%, and 7.06%, respectively (Grigorakis 2017).

**TABLE 5:** Fatty acid profile and lipid quality indexes in the fish muscle of Bartail flathead ( *Platycephalus indicus* ), Cleftbelly trevally ( *Atropus atropos* ), Japanese threadfin bream ( *Nemipterus japonicus* ), King soldier bream ( *Argyrops spinifer* ), and Pickhandle Barracuda ( *Sphyraena jello* ) (as % of total fatty acid), (mean ± SD; *n* = 3).

Fatty acids and lipid quality indexes	*Platycephalus indicus*	*Atropus atropos*	*Nemipterus japonicus*	*Argyrops spinifer*	*Sphyraena jello*
C8:0	1.19 ± 0.08^c,^ *	0.36 ± 0.08^d^	7.01 ± 0.24^b^	7.99 ± 0.26^a^	0.08 ± 0.03^d^
C10:0	1.03 ± 0.06^b^	0.38 ± 0.13^bc^	18.40 ± 0.72^a^	18.60 ± 0.88^a^	0.04 ± 0.01^c^
C12:0	0.80 ± 0.06^c^	0.10 ± 0.04^d^	18.60 ± 0.72^a^	15.33 ± 0.38^b^	0.16 ± 0.02^cd^
C14:0	7.41 ± 0.34^b^	7.14 ± 0.34^b^	2.62 ± 0.22^c^	2.08 ± 0.26^c^	8.45 ± 0.73^a^
C16:0	41.72 ± 0.92^b^	50.43 ± 0.79^a^	30.86 ± 0.88^d^	25.36 ± 0.41^e^	35.80 ± 0.96^c^
C16:1	7.80 ± 0.48^b^	9.21 ± 0.45^a^	4.32 ± 0.41^c^	7.35 ± 0.46^b^	9.49 ± 0.73^a^
C17:0	3.48 ± 0.18^ab^	3.03 ± 0.11^b^	4.43 ± 0.68^a^	3.58 ± 0.47^ab^	3.86 ± 0.88^ab^
C18:0	12.51 ± 0.63^a^	8.27 ± 0.67^b^	5.31 ± 0.53^d^	7.11 ± 0.18^c^	1.40 ± 0.44^e^
C18:1n9	16.23 ± 0.29^c^	17.77 ± 0.75^b^	8.64 ± 0.68^d^	9.90 ± 0.91^d^	25.34 ± 1.02^a^
C18:2n6	1.52 ± 0.24^a^	0.53 ± 0.06^b^	*	0.013 ± 0.01^c^	0.08 ± 0.01^c^
C20:0	0.60 ± 0.18^a^	0.26 ± 0.04^b^	0.04 ± 0.01^c^	0.013 ± 0.01^c^	0.02 ± 0.01^c^
C18:3n3	2.45 ± 0.53^a^	0.60 ± 0.08^c^	0.20 ± 0.04^c^	0.32 ± 0.08^c^	1.61 ± 0.47^b^
C22:0	0.59 ± 0.02^a^	0.15 ± 0.03^c^	0.02 ± 0.01^d^	0.20 ± 0.03^b^	0.13 ± 0.02^c^
C22:1n9	2.24 ± 0.32^b^	1.76 ± 0.30^b^	0.41 ± 0.08^c^	1.83 ± 0.50^b^	3.43 ± 0.69^a^
ƩSFA	69.32 ± 0.73^c^	70.12 ± 1.61^c^	87.39 ± 2.12^a^	80.26 ± 1.94^b^	49.92 ± 0.74^d^
ƩMUFA	26.27 ± 0.13^b^	28.74 ± 0.9^b^	13.37 ± 1.17^d^	19.07 ± 1.88^c^	38.26 ± 2.45^a^
ƩPUFA	3.97 ± 0.29^a^	1.13 ± 0.03^c^	0.20 ± 0.04^e^	0.33 ± 0.08^d^	1.69 ± 0.46^b^
Ʃω‐3	2.45 ± 0.53^a^	0.60 ± 0.08^c^	0.20 ± 0.04^c^	0.32 ± 0.08^c^	1.61 ± 0.47^b^
Ʃω‐6	1.52 ± 0.24^a^	0.53 ± 0.06^b^	*	0.01 ± 0.006^c^	0.08 ± 0.01^c^
PUFA/SFA	0.06^a^	0.02^b^	0.00^a^	0.003^a^	0.03^b^
ω‐3/ω‐6	1.68^b^	1.16^b^	0.00^b^	42.35^a^	21.08^ab^
AI	31.92 ± 1.45^ab^	30.46 ± 1.42^b^	31.45 ± 1.87^b^	25.09 ± 0.53^c^	34.92 ± 2.92^a^
TI	2.84 ± 0.25^b^	3.96 ± 0.37^a^	**	0.90 ± 0.67^c^	1.08 ± 0.22^c^
HH	0.41 ± 0.01^b^	0.33 ± 0.02^c^	0.26 ± 0.03^d^	0.37 ± 0.03^bc^	0.61 ± 0.01^a^
PI	7.02 ± 0.82^a^	2.40 ± 0.14^c^	0.73 ± 0.11^d^	1.09 ± 0.19^d^	4.17 ± 0.90^b^
HPI	109.36 ± 0.35^b^	411.72 ± 137.20^a^	42.08 ± 1.67^b^	34.94 ± 0.80^b^	316.74 ± 27.29^a^

*Note:* Different superscript letters (a–e) in the same row represent significant differences (*p* ≤ 0.05) among different fish species.

* Not detected.

** Not reported.

Blood cholesterol levels may rise if the PUFA/SFA ratio is less than 0.45 (Ivanova and Hadzhinikolova 2015). In the current study, the ratio of PUFA/SFA was varied between 0.003 to 0.06, and it was lower than the suggested standards. This indicates the poor lipid quality of the studied fish in the present study. Conversely, in the muscle of the *Anguilla anguilla* , the ratio of the polyunsaturated fatty acid to saturated fatty acid (PUFA/SFA) ranged from 0.48 to 0.52 (Gómez‐Limia et al. 2021). This ratio was also 1.90 and 0.60 in the *Oligoplites palometa* and *Seriola dumerili* , respectively (Fernandes et al. 2014). So, in the mentioned studies, PUFA/SFA was good. Foods high in PUFAs could be identified by their omega‐3 to omega‐6 (n‐3/n‐6) ratio (Gómez‐Limia et al. 2021). The ratio of n‐3/n‐6 is typically higher in fish lipids than in other meals, which is advantageous for the human diet (Usydus et al. 2011). In our study, the ratio of total omega‐3 to total omega‐6 (Ʃω‐3/Ʃω‐6) varied from 1.16 to 42.35. FAO/WHO suggested an n‐3/n‐6 ratio between 1–8 and 2–5 (FAO/WHO 2003). This ratio in *P. indicus* (1.68) and *A. atropos* (1.16), the value of this ratio was within the advised range. Conversely, while in *A. spinifer* and *S. jello* , it was very high, which is probably because the only omega‐6 detected was linoleic acid and its levels are very low (0.13% and 0.08%), respectively. This ratio varied from 1.13 to 2.15 in *Liza aurata* and from 0.81 to 1.13 in *Mulloidichthys vanicolensis* (Küçükgülmez et al. 2018).

The atherogenic index (AI), thrombogenic index (TI), and the lipid quality indexes for fish species in this study are displayed in Table 5. In this study, the quality of muscle lipids based on the found AI range (34.92 to 25.09) was detrimental to cardiovascular health. The TI values of the five species ranged from 0.9 to 3.96; only *A. spinifer* had a favorable status (90 ± 0.67), and the intake of its lipid is good for cardiovascular health. The good quality lipid of *Anguilla anguilla* has been confirmed by its AI and TI, which varied across different weight ranges from 0.64 to 0.66 and 0.58 to 0.64, respectively (Gómez‐Limia et al. 2021). According to Araujo et al. (2021), the lipid quality indices AI and TI for *Seriola dumerili* and *Trematomus bernacchii* were 0.82, 0.54, 0.29, and 0.22, respectively; hence, both fish had good status based on these indices. The relation between total unsaturated and total saturated fatty acids is shown in the AI. According to Yurchenko et al. (2018), consuming foods with low AI decreases the total cholesterol levels of blood plasma in humans. The relationship between prothrombogenic fatty acids (lauric, myristic, and palmitic acids) and anti‐thrombogenic fatty acids (monounsaturated fatty acids, omega‐3 and omega‐6 PUFAs) is represented by the TI, which indicates the propensity for clot formation in blood vessels; therefore, consuming foods with low TI levels is good for the cardiovascular system (Ulbricht and Southgate 1991). In the present study, the HH ratio ranged from 0.26 to 0.61, indicating the lipids of these fish had weak health‐promoting effects. In contrast, the results of the study by Rincón‐Cervera et al. (2020) showed that the HH ratio examined species was favorable. In the mentioned study, the HH values of yellowtail amberjack ( *Seriola lalandi* ), red hake ( *Genypterus chilensis* ), red mullet ( *Otolithes ruber* ), mackerel ( *Scomber scombrus* ), Chilean hake ( *Merluccius gayi* ), and jack mackerel ( *Trachurus symmetricus* ) were 2.14, 2.93, 1.86, 2, 2.23, and 1.73, respectively. A higher value of the HH is used to assess the nutritional benefits of lipids in food products. Foods with high HH values are considered high lipid quality foods, since it has beenstated that a HH a value higher than 2 has positive health effects (Santos‐Silva et al. 2002). In the current study, the PI ranged from 0.73 to 7.02. The PI value of *Platycephalus indicus* (7.02) was significantly (*p* < 0.05) higher than other fish. Similarly, lower PI values were reported for *Liza aurata* (0.32 to 0.55), *Diplodus vulgaris* (0.50 to 0.56) (Küçükgülmez et al. 2018), and anchovy oil (1.33 to 1.41) (Bayraklı and Duyar 2019). The stability of PUFA in food and their oxidation resistance is assessed using the fatty acid oxidation potential (PI) index (Zula and Desta 2021). A great potential for prevention against cardiovascular illnesses is indicated by the high values of this index (Kang et al. 2005). In this study, *A. spinifer* (34.94) and *A. atropos* (411.72) had the lowest and highest HPI values, respectively. Contrary to our study, the amount of this index was reported at low levels for golden gray mullet (0.32–0.55) and gold band goatfish (0.50–0.56) (Küçükgülmez et al. 2018) and in anchovy oil (1.33–1.41) (Bayraklı and Duyar 2019). The impact of fatty acid content on cardiovascular illnesses is the main focus of the HPI, which has been developed to assess the nutritional value of dietary lipids (Karsli 2021). It has been stated that high HPI is helpful for human health (Chen et al. 2004). Thus, based on this ratio, the lipid of *A. atropos* in comparison with other species is more beneficial for the health of humans.

### 3.5 Trace Elements

Table 6 demonstrates the results of the trace elements study of fish. In addition, Table 6 presents the recommended daily intake for an adult for each element suggested by the National Research Council, Commission on Life Sciences, and Subcommittee on the Tenth Edition of the Recommended Dietary Allowances (1989). Generally, the concentrations of macroelements in the five species examined occurred in the following sequence: K > Ca > Na > Mg > P. In addition, a significant difference (*p* ≤ 0.05) was recorded among five species for all macroelement concentrations (Table 6). Potassium (K) first abundant macroelement in all the samples examined, so the highest K concentration (13,578.73 μg/g) was found in *S. jello* . This macroelement is an essential dietary electrolyte that plays a central role in the preservation of membrane potential and operates as a cofactor for some enzymes (Sarojnalini and Hei 2019). Inconsistent with this study, in Bogue fish ( *Boops boops* ) amount of K during the year was 934, 3386, 2080, and 374 in Winter, Spring, Summer, and Autumn, respectively (Uçar 2020). Based on the daily recommended demand for adults by FAO/WHO (Table 6) to meet daily K requirements, 257 g of *S. jello* muscle is needed (FAO/WHO RDI). The second abundant macroelement was calcium. The highest calcium (Ca) concentration (7212.16 μg/g) was obtained in *A. atropos* . Unlike this study, low concentrations were reported in some marine fish; for example, Moxness Reksten et al. (2020) reported that Ca content in *Caranx rhonchus* , *Sardinella aurita* , *Sardinella maderensis* , *Sardinella guachancho*, and *Trachurus trecae* was 25.8, 71.2, 89.6, 20.4, and 24.6 mg/100 g, respectively. The finding of our study shows that an intake of 111 g *A. atropos* muscle can provide the daily demand of an adult for this microelement.

**TABLE 6:** Trace elements content (μg/g dry sample) and some ratios in the fish muscle of Bartail flathead ( *Platycephalus indicus* ), Cleftbelly trevally ( *Atropus atropos* ), Japanese threadfin bream ( *Nemipterus japonicus* ), King soldier bream ( *Argyrops spinifer* ) and Pickhandle Barracuda ( *Sphyraena jello* ), (mean ± SD; *n* = 3).

Trace elements	*Platycephalus indicus*	*Atropus atropos*	*Nemipterus japonicus*	*Argyrops spinifer*	*Sphyraena jello*	LOD	LOQ	Daily demand for adults
Microelements								
Na	3587.04 ± 433.16^a^	2655.64 ± 344.55^b^	2891.18 ± 220.49^b^	2818.87 ± 228.79^b^	3033.61 ± 640.95^b^	1.10	3.24	500*
Mg	1161.17 ± 101.18^b^	1014.74 ± 48.45^c^	1403.44 ± 32.81^a^	1240.00 ± 102.54^b^	1291.43 ± 191.16^ab^	0.56	1.75	280–350
K	8856.29 ± 1085.25^b^	7530.98 ± 392.46^b^	11,695.83 ± 1231.38^a^	12,401.71 ± 551.65^a^	13,578.73 ± 2830.57^a^	1.02	3.01	3500
Ca	1869.21 ± 780.21^b^	7212.16 ± 4797.43^a^	3269.10 ± 2368.21^b^	3602.92 ± 888.75^b^	1629.11 ± 974.36^b^	1.50	5.55	800
P	730.11 ± 26.58^c^	835.84 ± 30.03^b^	867.93 ± 22.37^b^	867.38 ± 11.70^b^	942.39 ± 23.84^a^	2.35	7.00	800
Microelements								
Mn	2.24 ± 2.25^a^	1.66 ± 1.14^a^	1.66 ± 0.87^a^	1.08 ± 0.42^a^	1.09 ± 0.56^a^	0.01	0.03	2–5
Fe	42.20 ± 13.93^a^	29.26 ± 4.45^a^	55.48 ± 40.09^a^	40.14 ± 14.07^a^	36.59 ± 13.06^a^	0.05	0.18	10
Cu	2.76 ± 0.78^a^	1.29 ± 0.23^a^	2.06 ± 0.92^a^	1.79 ± 0.33^a^	5.05 ± 5.14^a^	0.01	0.04	1.5–3
Zn	24.96 ± 14.25^a^	12.59 ± 1.26^a^	13.93 ± 3.33^a^	27.07 ± 23.86^a^	14.02 ± 5.10^a^	0.02	0.05	12–15
Se	3.93 ± 0.39^a^	2.54 ± 0.55^b^	3.62 ± 0.2^a^	3.90 ± 0.29^a^	3.03 ± 0.48^b^	0.01	0.03	55–70
Mo	2.05 ± 1.71^a^	0.19 ± 0.09^a^	0.24 ± 0.20^a^	2.18 ± 3.97^a^	0.58 ± 0.45^a^	0.01	0.03	
Ratio								
Na/K	0.41	0.35	0.25	0.23	0.22			
Ca/P	2.57	8.63	3.74	4.15	1.73			
Ca/Mg	1.60	7.05	2.33	2.92	1.24			
Ca/K	0.21	0.97	0.30	0.29	0.12			

*Note:* Different superscript letters (a–c) in the same row represent significant differences (*p* ≤ 0.05) among various fish species.

* Daily demand adult to trace elements includes Sodium (Na), Magnesium (Mg), Potassium (K), Calcium (Ca), phosphor (P), Manganese (Mn), Iron (Fe), Copper (Cu), Zink (Zn) as mg/day and for Selenium (Se) and Molybdenum (Mo) as μg/g/day (National Research Council, Commission on Life Sciences, and Subcommittee on the Tenth Edition of the Recommended Dietary Allowances 1989). The intake amount of sodium in the human diet is lower than 2 g/day, was suggested, approximately 5 g of sodium chloride salt.

Microelements are required in small amounts; however, these nutrient compositions are essential for proper growth and development, as well as their deficiency being a type of malnutrition and a health problem in many developing countries (Mohanty et al. 2016). In the present study, there was no significant difference (*p* > 0.05) in the concentrations of manganese (Mn), iron (Fe), copper (Cu), zinc (Zn), and molybdenum (Mo) in the muscles of the five studied species. The concentrations of macro elements in the five species examined occurred in the following sequence: Fe > Zn > Se > Cu > Mn > Mo. Fe was the first abundant microelement, and the highest Fe concentration (55.48 μg/g) was measured in *N. japonicus* . Fe is required for the synthesis of amino acids, collagen, neurotransmitters, neuronal development, cellular function, and hormone synthesis (Tacon et al. 2020). Based on this study, an intake of 180 g of *N. japonicus* muscle provides the daily demand for an adult for this element. Zn was the second abundant microelement, and the highest Zn concentration (27.07 μg/g) was measured in *A. spinifer* . Zn is involved in cell proliferation and gene expression, nucleic acid and amino acid metabolism, and the metabolism of vitamins A and E (Prasad 2014). An intake of 443 g of *A. spinifer* muscle provides the daily demand for an adult for this microelement. The Se concentration in *P. indicus* , *N. japonicus* , and *A. spinifer* was higher than that in *A. atropos* and *S. jello* significantly (*p* ≤ 0.05). The highest Se (3.93 μg/g) was obtained in *P. indicus* . Soltani et al. (2021) showed that Se concentration, unlike our study, was low; thus, this essential element in the muscle of *Euryglossa orientalis* , *Sardinella longiceps* , and *Carcharhinus dussumieri* was 0.54, 0.21, and 0.95 mg/kg. Se acts as an antioxidant and catalyst for the production of thyroid hormone and stimulates immune system function (Rayman 2000). Based on the results of this study, consuming 15.9 g of *P. indicus* muscle *is* suggested to supply the daily demand for an adult for this microelement. Mineral ratios (e.g., Na/K, Ca/P) help diagnose nutrient imbalances or metabolic risks (Adeyeye et al. 2019). Understanding the Na/K ratio is crucial for preventing hypertension and atherosclerosis in the human body (Cook et al. 2009). The recommended amount for this ratio is less than 0.5 (WHO 2012). The Na/K was 0.22 to 0.41 (Table 5), within the WHO range. The Na/K ratio for both the common shrimp ( *Palaemon serratus* ) and the Atlantic ditch shrimp ( *Palaemon varians* ) was 0.57 and 0.67, which was somewhat higher than the recommended value (Maia et al. 2022). A food with a ratio of Ca/P more than one is considered good, and poor if the ratio is less than 0.5 (Aremu et al. 2013). The Ca/P ratio in this study ranged from 1.73 to 8.63 (Table 5). This ratio was higher than the suggested value, which indicates the favorable status of the studied fish species. The ratio of Ca/P in the backbones of codfish and Atlantic salmon is 2.07 and 1.99, respectively (Bubel et al. 2015). The ratio of Ca/Mg in the range of 2 to 16 is advised for emotional and mental stability; in contrast, emotional and mental disorders may result from values higher than 16 or less than 2 (Adeyeye et al. 2019). The Ca/Mg was less than 2 in *P. indicus* (1.60) and *S. jello* (1.24), and more than 2 in *A. atropos* , *N. japonicus* , and *A. spinifer* . The ratio of Ca/Mg in *Alburnus chalcoides* was 2.74 (Subotić et al. 2021). Since Ca and K are essential for controlling thyroid action, the Ca/K is frequently called the thyroid ratio. As well, the range of 5–7 for this ratio is necessary to preserve thyroid action regulation and establish a healthy equilibrium (Aremu et al. 2013). In this study, this ratio ranged from 0.12 to 0.97, which was an undesirable value. Differences in mineral concentrations of fish are attributed to seasonal and biological variables, including species, size, age, sex, dark or white muscle, food source, and environmental states like salinity, temperature, and pollutants (Alasalvar et al. 2002). Benthic species ( *P. indicus* , *A. spinifer* ) accumulated higher Na/Mg, while pelagic *S. jello* 's active metabolism may explain elevated P/K levels (Merciai et al. 2014). The benthic nature of *P. indicus* and *A. spinifer* , as well as the high metabolic rate in the *S. jello* , may be responsible for the high Na levels in *P. indicus* , the high Mg levels in *A. spinifer* , and the high P and K levels in *S. jello* .

## 4 Conclusion

This study presents the first comprehensive nutritional evaluation of five commercially important fish species from the Persian Gulf. All species represent excellent low‐calorie protein sources (108–126 kcal/100 g) with favorable Na/K ratios (0.22–0.41). *N. japonicus* was identified as the superior protein source (EAAI: 75.92; BV: 71.02), while *P. indicus* demonstrated the most favorable lipid profile (PUFA: 3.97%; PI: 7.02). Practical nutritional benefits were evident, with 100–150 g portions providing 25%–50% of daily requirements for key minerals (K, Ca, Fe, Zn). The exceptional lysine content (up to 12.37 g/100 g) further establishes these species as valuable resources for regional dietary strategies and public health nutrition programs. The highlight of the current study's limitations was the sampling season, high diversity of edible fish, and limitations in analyzing the large number of commercial species. In addition to this, the authors' suggestions for future studies are to select fish species from other commercial fish families in the Persian Gulf and in all seasons of the year for a better understanding of changes in nutritional compositions.

## Author Contributions

**Behrooz Mohammadzadeh:** conceptualization, methodology, investigation, writing‐original draft, formal analysis. **Javad Feizy:** conceptualization, methodology, data curation, review, and editing.

## Ethics Statement

All sampling and handling processes in the present study were performed under applicable international, national, and/or institutional guidelines for the care and use of animals.

## Conflicts of Interest

The authors declare no conflicts of interest.

## Data Availability

The data that support the findings of this study are available on request from the corresponding author.
